# MicroRNA-9 restrains the sharp increase and boost apoptosis of human acute myeloid leukemia cells by adjusting the Hippo/YAP signaling pathway

**DOI:** 10.1080/21655979.2021.1915727

**Published:** 2021-06-24

**Authors:** Guoqing Wang, Xiuwen Yu, Jiajia Xia, Jie Sun, Haiyan Huang, Yeqiong Liu

**Affiliations:** Department of Clinical Laboratory, Jiading District Central Hospital Affiliated to Shanghai Health Medical College, Shanghai, China

**Keywords:** mir-9, Hippo/YAP signaling pathway, acute myeloid leukemia, proliferation and apoptosis

## Abstract

MicroRNAs (miRNAs) play a very important role in the development of acute myeloid leukemia (AML). This study focuses on the effects of miR-9 on the regulation of AML cells and their related signaling pathways. We found that the expression of miR-9 was significantly decreased in four AML cell lines (THP-1, HL-60, TF-1 and KG-1) compared with the human normal bone marrow cells (HS-5). Moreover, miR-9 overexpression inhibited HL-60 cell proliferation ability, and promoted apoptosis. However, interfering with miR-9 expression promoted the proliferation of HL-6 cells and inhibited apoptosis. Western blotting results subsequently showed that overexpression of miR-9 could elevate the expression of MAT1, LATS1, and LATS2 in HL-60 cells, and inhibit the expression of YAP, while the interference with miR-9 had the opposite result. Taken together, miR-9 may act as a tumor suppressor by activating the Hippo/YAP signaling pathway of AML cells, which in this way supply ideas for the clinical remedy of AML patients.

## Graphical abstract

**Figure:**
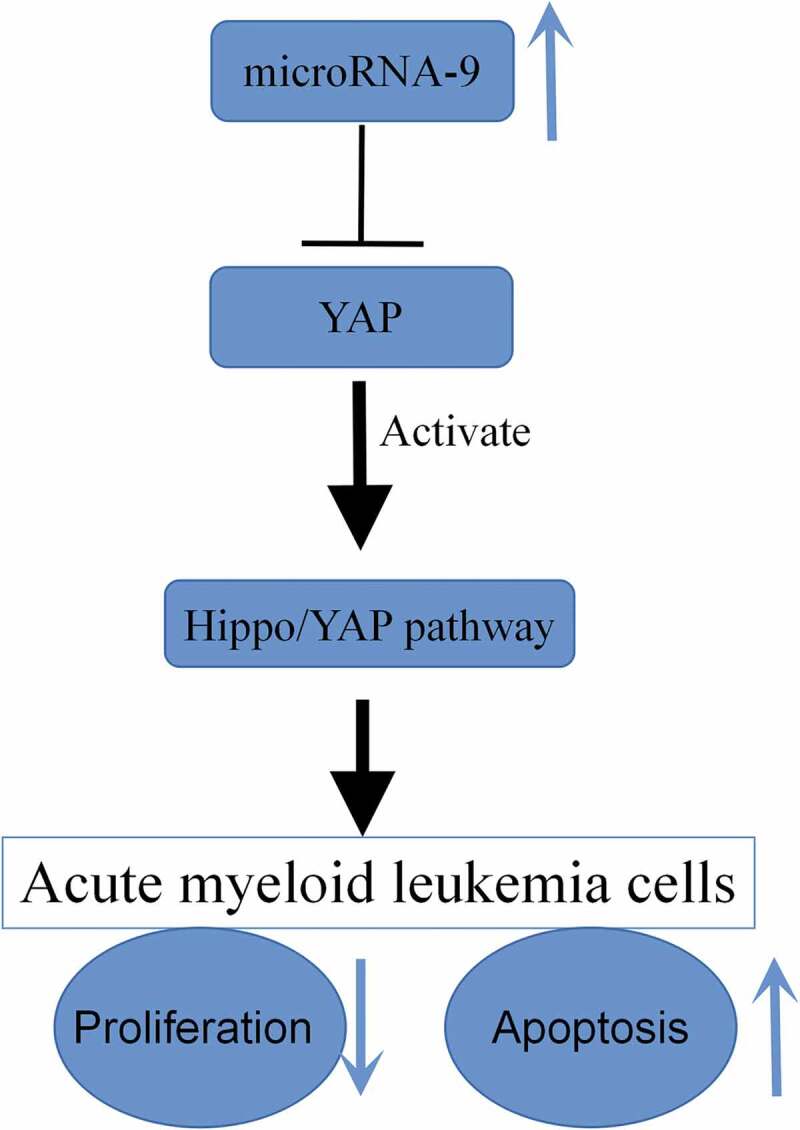

## Introduction

Acute myeloid leukemia (AML) is a highly heterogeneous hematological malignant tumor [1]. Its occurrence is associated with complex and variable genetic mutations [2]. AML is a shared type of leukemia, accounting for about 33% of all types in the United States, and the incidence of acute myeloid leukemia in China ranks third in the world. Among young patients who suffer from acute myeloid leukemia, China ranks highest in mortality rate [3]. The main treatment methods are hematopoietic stem cell transplantation and chemotherapy, but only a few people can complete hematopoietic stem cell transplantation so the overall five-year survival rate is less than 20%, and the results are not satisfactory. The incidence of AML is higher in men than women, and as the age increases, the incidence of AML also gradually increases. Although the progress of supportive treatment and prognostic risk stratification has optimized the established treatment method, the overall long-term survival rate is still very low [4]. Therefore, it is practically momentous to find an effective target that can early diagnose and treat AML.

MicroRNA (miRNA) is a type of endogenous small-molecule RNA, about 20–24 nucleotide residues in length, which is widely present in various organisms [5]. miRNA has no translational effect, but affects gene expression, regulates the cell cycle and individual development, and is very vital in the course of sharply increasing differentiation and apoptosis of cell [6]. The latest research has attested that miRNAs are latent and prospective tools for cancer diagnosis and remedy [7]. It has been reported that miRNAs are significantly imperative in the occurrence, development and prognosis of AML [8]. Rashed et al. [9] confirm that miR-92a can be used as a marker of treatment response and survival in adult patients with AML. Wang et al. [10] reveal that miR-582-5p can inhibit the proliferation and induce apoptosis in AML cells. Of late, abnormal expression of miR-9 has been found in many tumor-related studies, which may be related to the development of tumors. miR-9 expression is enhanced in children and adults with acute leukemia, and miR-9 is closely related to leukemia, according to the findings of some studies [11]. Therefore, miR-9 may play a remarkably vital role in the emergence and growth of leukemia. However, the function of miR-9 in regulating AML development and progression is still unknown.

Like many tumor cells, the occurrence and development of AML are related to many signaling pathways, including Hippo/YAP. Studies at home and abroad have confirmed that miR-7977 can inhibit the proliferation of bone marrow mesenchymal stromal cells through the Hippo/YAP signaling pathway [12]. Recently, there were also reports in the literature that breast cancer dryness is promoted by miR-520b through the pathway Hippo/YAP [13], and miR-375 mediates Hippo/YAP pathway involvement in gastric carcinoma occurrence by hitting the YAP1/TEAD4-CTGF axis [14]. These miRNAs are able to biologically function in neoplastic diseases through Hippo/YAP. Therefore, we inferred that miR-9 may also have an impact on the biological functions such as the proliferation of AML cells through the Hippo/YAP signal pathway. This study will be the first to carry out the hypothesis so as to provide new ideas for the treatment of AML.

## Materials and methods

### Cell culture

Human normal bone marrow cells HS-5, AML cell lines THP-1, HL-60, TF-1 and KG-1 were obtained from the Cytology Library of the Chinese Academy of Sciences. All the cells were cultured in DMEM containing 10% fetal bovine serum (FBS, Invitrogen), 25 μg/ml penicillin, and 25 μg/ml streptomycin (Invitrogen). The process of culture was at 37°C in a 5% CO_2_ constant temperature cell incubator.

### Cell transfection

miR-9 mimic, NC mimic, miR-9 inhibitor, and NC inhibitor, obtained from RiboBio (Guangzhou, China), were transfected with Lipofectamine 2000 strictly according to the reagent instructions provided. HL-60 cells were split into five groups: miR-9 mimic group, overexpression transfection-negative control group (NC mimic), miR-9 inhibitor group, interference transfection-negative control group (NC inhibitor), and untransfected group (Sham group). HL-60 cells were seeded on a 6-well plate at a concentration of 5 × 10^5^ cells/well. After transfection for 6 h, they were washed three times with PBS and replaced with ordinary culture medium for 48 h. Transfection efficiency was verified by qRT-PCR.

### Cell viability assay

With miR-9 mimic, NC mimic, miR-9 inhibitor cells were, respectively, transfected, and NC inhibitor. After infection culture overnight, the culture medium was replaced after transfection culture for 24 h, 48 h, and 72 h, 10 μL CCK-8 and 90 μL DMEM medium containing 10% FBS per well. After being incubated in the incubator for 1 h, the absorbance at 450 nm wavelength was determined by multi-function enzyme labeling instrument.

### Clone formation assay

After 48 hours of transfection, HL-60 cells were prepared into a single-cell suspension, and 3000 cells were inoculated in 6-well plates. The cells were put into an incubator at 37°C and with 5% for 2 weeks, and the culture was terminated when the cell clones were visible. The upper culture solution was discarded, and cells were fixed with paraformaldehyde for 15 min. Then, the cells were stained with crystal violet for 5 min. At last, the number of cell clones was recorded.

### Flow cytometry

The operation of flow cytometry to detect apoptosis was carried out according to the kit instructions (Biyuntian, Shanghai). After 48 h of transfection, the cells in each group were washed with PBS for 2 times. After centrifugation, the cells were re-suspended with 100 μl 1× binding buffer; then, 5 μl Annexin V-FITC and 5 μl PI staining solution were put into gently mixed and incubated without light for 15 min. At last, cells were caught on the machine for testing. The apoptosis rate was gauged by FACS-Calibur flow cytometry. The experiment was done in triplicate.

### Quantitative RT-PCR

RNA of cells was completely obtained by Trizol reagent in the light of the steps in instructions. According to the steps in the instructions of the TaqMan MicroRNA Reverse Transcription Kit, 2 μg of total RNA were reversely transcribed into cDNA, respectively. Two μL cDNA and 4 μL forward primers sequence and 4 μL reverse primer sequences were selected to carry out RT-qPCR reaction with the ABI PRISM 7500 PCR instrument according to the instructions of the analysis kit. The following forward and reverse primers were used: miR-9: 5ʹ-GCGCTCTTTGGTTATCTAGCT-3ʹ and 5ʹ-GCGCTGCAGGGTCCGAGGT-3ʹ; U6: 5ʹ-GCTTCGGCAGCACATATACTAAAAT-3ʹ, and 5ʹ-CGCTTCACGAATTTGCGTGTCAT-3ʹ. Pre-denaturation at 94°C for 30 min and then 40 cycles (95°C 5 s, 60°C 30 s, 74°C 30 s) were the reaction conditions. Using U6 as the internal control of miR-9, the relative expression quantity of miR-9 was calculated by 2^−ΔΔCt^ method.

### Western blot

Proteins were isolated from transfected cell. The protein concentration was then detected by the BCA method. The protein was separated by 12% SDS-PAGE. After electrophoresis, the proteins were transferred to PVDF membranes and then blocked with 5% nonfat milk for 1 h at room temperature. The membrane was incubated with antibody MAT1 (1:2000 dilution, ab129176), LATS1 (1:1000 dilution, ab70561), LATS2 (1:2000 dilution, ab110780), YAP (1:1500 dilution, ab205270), and β-actin (1:1000 dilution, ab179467) overnight at 4°C. Then, the corresponding secondary antibody (diluted at 1:4 000) was appended in the proteins and cultured at 37°C for 1 h. They were immersed in ECL solution in the darkroom for color development, and the film exposure was carried out. Using β-actin as the internal reference of the target band, Quantity One software analyzed the relative expression quantity of the protein band.

### Statistical analysis

All data were expressed as mean ±standard deviation (SD) of at least three experiments. SPSS 19.0 was used to carry out statistical analysis. Student’s t-test or one-way ANOVA was used to evaluate the difference between two groups or more than two groups, respectively. *P* < 0.05 was considered statistically significant.

## Results

### miR-9 was lowly expressed in AML cell lines

**Figure 1.**
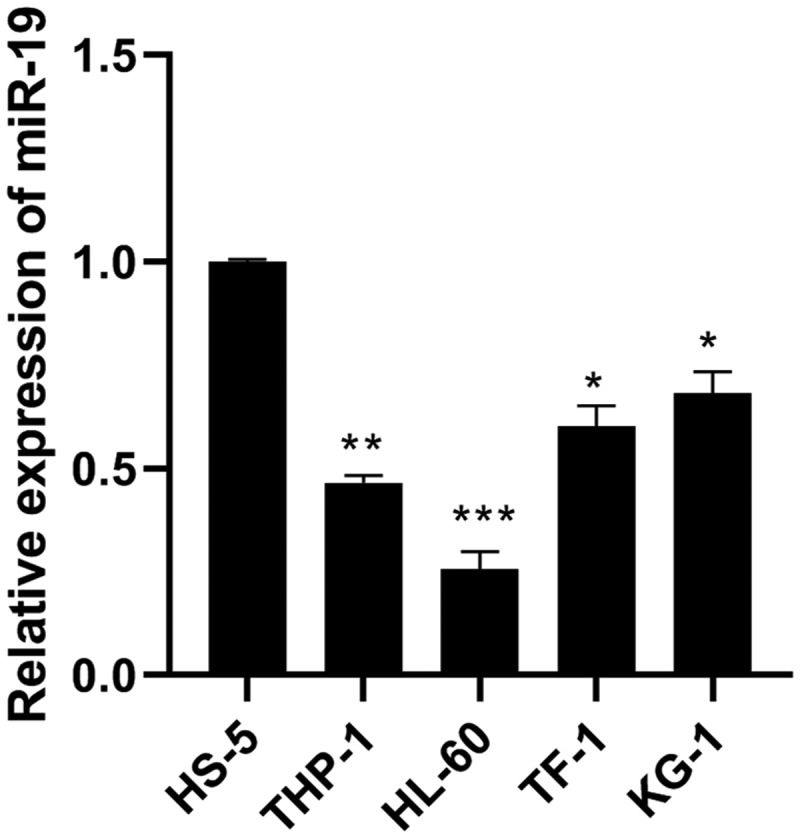
The expression of miR-9 was downregulated in AML cell lines. Relative expression of miR-9 was measured in AML cell lines (THP-1, HL-60, TF-1 and KG-1) and human normal bone marrow cells (HS-5) using qRT-PCR. *P < 0.05, **P < 0.01, ***P < 0.0001 vs. HS-5 cell

Compared with human normal bone marrow cells (HS-5), the expression of miR-9 in AML cancer cell lines THP-1, HL-60, TF-1 and KG-1 was significantly reduced (Figure 1), and the expression of miR-9 in HL-60 decreased most obviously, so HL-60 was used for subsequent experiments.

### Biological effect of miR-9 in inhibiting proliferation and promoting apoptosis of AML cells

**Figure 2.**
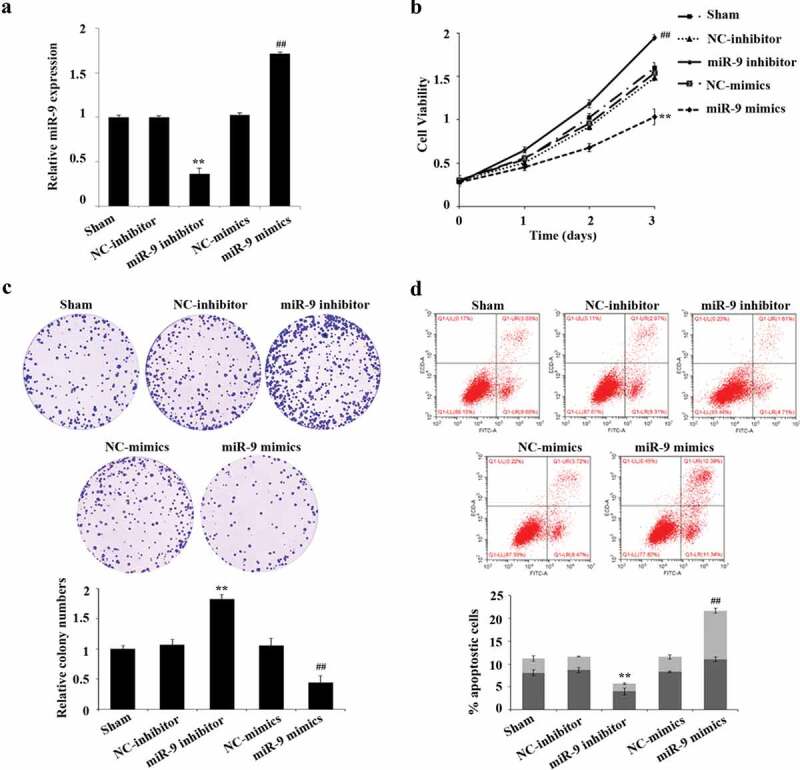
miR-9 restrain the sharp increase of AML cells and boost their apoptosis. After transfection of miR-9 mimic, NC mimic, miR-9 inhibitor and NC inhibitor, QRT-PCR was used to find out the expression of miR-9; (b) after transfection of miR-9 mimic, NC mimic, miR-9 inhibitor and NC inhibitor, CCK-8 was used to detect the cell viability of HL-60. (c) After transfection of miR-9 mimic, the abrupt increase of HL-60 cells was detected by cell colony assay, NC mimic, miR-9 inhibitor and NC inhibitor; (d) After transfection of miR-9 mimic, NC mimic, miR-9 inhibitor and NC inhibitor, flow cytometry was used to detect the apoptosis rate of HL-60. Compared with NC inhibitor, **P < 0.01; compared with NC mimic, ^##^*P* < 0.01

In order to explore the biological effect of miR-9 in AML cells, we conducted function gain and loss experiments by overexpressing or the expression of miR-9. MiR-9 mimic, NC mimic, miR-9 inhibitor were interfered and the NC inhibitor was transfected in HL-60 cells. The results of qRT-PCR verification manifested that miR-9 inhibitor can significantly reduce the expression of miR-9 (P < 0.05), while miR-9 mimics can increase the expression of miR-9 (P < 0.05) (Figure 2a).

After successful transfection, we used CCK-8, plate cloning formation assay, and flow cytometry to detect the cell viability, cell proliferation, and apoptosis of HL-60, respectively. The results showed that compared with the NC inhibitor group, the cell proliferation in the miR-9 inhibitor group was significantly increased, and the apoptosis rate was significantly reduced (P < 0.05); the cell proliferation in the miR-9 mimic group significantly decreased compared with the NC-mimic group, the apoptosis rate was significantly risen (P < 0.05) (Figure 2b–Figure 2d).

### The effect of miR-9 on Hippo/YAP signaling pathway in HL-60 cells

**Figure 3.**
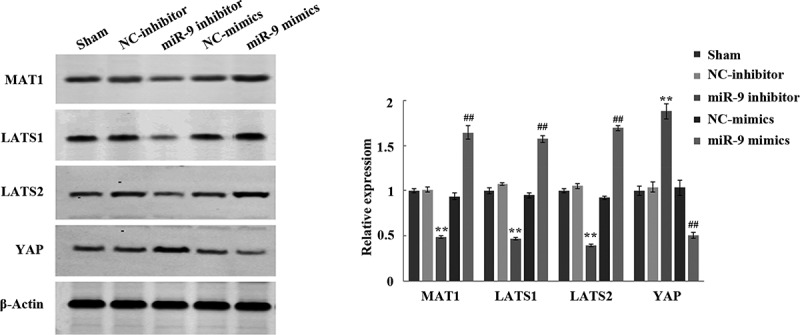
Effect of miR-9 on Hippo/YAP signal pathway in HL-60 cells. After transfection of miR-9 mimic, NC mimic, miR-9 inhibitor, and NC inhibitor, the expression levels of the corresponding proteins MAT1, LATS1, LATS2, and YAP in the Hippo/YAP signaling pathway in HL-60 cells was detected by using the Western blot

In order to verify whether miR-9 affects biological functions such as sharp increase and apoptosis of AML cells by inhibiting Hippo/YAP signaling pathway, after transfection of miR-9 mimic, NC mimic, miR-9 inhibitor, and NC inhibitor, the expression levels of the corresponding proteins MAT1, LATS1, LATS2, and YAP in the Hippo/YAP signaling pathway were tested. Western blot experiments showed that, the protein expression levels of MAT1, LATS1, and LATS2 in the miR-9 inhibitor group were significantly reduced, and the expression level of YAP was significantly increased (P < 0.05) compared with the NC inhibitor group; the protein expression levels of MAT1, LATS1, and LATS2 in the miR-9 mimic group increased significantly compared with the NC mimic group, while the expression level of YAP decreased significantly (P < 0.05) (Figure 3). This result suggested that miR-9 mediates biological functions of AML cell apoptosis by activating the Hippo/YAP signaling pathway.

## Discussion

Despite the great progress in the treatment of AML, there are still quite a few patients with a poor prognosis, so it is of great significance to find new therapeutic targets for AML [15]. In recent years, it has been found that miRNA is in a close relationship with the occurrence and growth of leukemia, and it may be significant in the occurrence of AML by monitoring the corresponding signaling pathways [16]. SM Gao et al. reached a conclusion that miR-15a/miR-16-1 was reduced in most acute B-lymphocytic leukemia. The reduced miR-15a/miR-16-1 induced leukemia cell apoptosis by targeting Bcl-2 plays the role of tumor suppressor genes [17]. As Wang et al. informed, in the differentiation of myeloid cells miR-29a and miR-142-3p are significant, they regulated granulocyte differentiation [18]. Thus, it is valuable to find AML-related miRNA and further study its function and regulatory mechanism, which is not only of great significance to reveal the ‘RNA regulatory network’ in leukemia, but also may provide a new target for miRNA to be used in the treatment of AML.

With the continuous discovery of disease-related miRNA, miRNA is biologically important in the tumor respects [19]. miR-9 is usually abnormally expressed in a variety of cancers and takes part in many kinds of biological courses, including sharp increase, shift and intrusion [20]. For example, Chen et al. affirmed that restraining the level of miR-9 in prostate cancer can exert a carcinogenic effect by targeting StarD13 [21]. Dong et al. discovered that down-regulation of miR-9 in bladder cancer may regulate cell cycle arrest and apoptosis [22]. Chen et al. obtained a result that down-regulating BRAF expression helped miR-9-5p increase the sensitivity of thyroid cancer cells to cisplatin [23]. Recently, some studies have displayed that miR-9 also biologically functions in the progression of AML. For example, Liu et al. presented that the expression of miR-9 is down-regulated in AML patients, with low overall survival and poor progression-free survival [24]. Nishioka et al. reached a conclusion that miR-9 partakes in the expression of E-cadherin of interleukin 10 mediated in acute myeloid leukemia cells [25]. Chen et al. saw that miR-9 regulates the sharp increase in normal karyotype adult CD34 positive AML cells by down-regulating Hes1 [26]. However, some issues regarding the mechanism of action of miR-9 in AML remain unclear. In our current study, we first ascertained that miR-9 is lowly expressed in bone marrow tissues and cell lines of AML patients. Further, through function gain and deficiency experiments, it was confirmed that overexpression of miR-9 can significantly restrain AML cell sharp increase and boost cell apoptosis in vitro. This result suggested that miR-9 can significantly restrain the emergence and growth of AML.

The physiological courses of tumor cell growth, sharp increase, differentiation, apoptosis, shoft, intrusion and angiogenesis were cross-regulated by the Hippo/YAP signaling pathway [27], one of the multiple signaling pathways. Found in Drosophila, Hippo/YAP signaling pathway is a cell signal transduction pathway. The Hippo/YAP signaling pathway in the human body can negatively regulate its downstream transcription coactivator Yes-related protein (YAP) to inhibit cell growth, induce apoptosis, and accommodate organ size. Once the Hippo/YAP signaling pathway is inactivated, it will induce the activation of multiple target genes mediated by YAP and regulate the growth, proliferation, invasion and apoptosis of cancer, thus affecting the treatment and prognosis of patients [28]. The pathway is mainly comprised of Hippo (Hpo), Salvador (Sav), Warts (Wts), Yorkie (Yki), etc. In mammals, it is mainly composed of Mst1/2, WW45, LATS1/2, MAT1/2 and YAP. The activation of the Hippo/Salvador kinase complex in this pathway is regulated by Cytoskeletal connexins Merlin and Expanded. The Warts/Mats kinase complex and Warts/Mats kinase phosphorylates are mainly energized by the latter, and the downstream transcription coactivator Yki is restrained, which positively monitors cell growth, survival, and sharp increase.

A large number of studies have verified that overexpression of YAP in the Hippo/YAP signaling pathway can induce epithelial–mesenchymal transition (EMT) and is independent of growth factors and supports. YAP is amplified in 11q22 of human chromosomes and has a carcinogenic effect. The EMT transcription factor Zeb1 is jointly regulated by TAZ and its activator Tead1, TAZ and its activator Tead1 can also promote cell proliferation and the occurrence of EMT. TAZ’s key members, MAT and LATS, can phosphorylate and inactivate TAZ, thereby inhibiting cell proliferation and the occurrence of EMT, indicating that Hippo/The YAP signaling pathway can inhibit the occurrence of EMT and enhance the suppression of contact between cells [29]. Furthermore, YAP is also one kind of gene among downstream target genes of miR-9 [30,31]. Therefore, we speculated that miR-9 inhibits the occurrence and development of AML, which is closely related to the Hippo/YAP signaling pathway. In this study, we continued to transfect miR-9 mimic and inhibitor into HL-60 cells, and observed their effects on the inactivation of Hippo/YAP signal pathway. Restraining the expression of miR-9, as the results presented, could importantly reduce the protein expression level of MAT1, LATS1, LATS2 and increase the expression level of YAP, whereas the results are just the contrary in terms of miR-9 mimic. These data indicated that miR-9 can energize the Hippo/YAP signaling pathway in HL-60 cells, thereby regulating the expression of MAT1, LATS1, LATS2 and YAP, and ultimately restraining cell sharp increase and lead to apoptosis.

## Conclusion

In conclusion, our study revealed the low expression of miR-9 in AML. Overexpression of miR-9 inhibited the proliferation of HL-60 cells, promoted their apoptosis, and activated the Hippo/YAP signaling pathway, thereby slowing down the development of AML. The discovery of these results is significant for miR-9 to become a useful marker and underlying therapeutic target for the diagnosis and treatment of AML.

## Supplementary Material

Supplemental Material

## Data Availability

All data, models, and code generated or used during the study appear in the submitted article.

## Highlights

1. The expression of miR-9 is significantly decreased in AML cancer cell lines.
2. miR-9 overexpression inhibits HL-60 cell proliferation and promoted cell apoptosis.
3. miR-9 overexpression activates the Hippo/YAP signaling pathway.
