# Supplementary figures and images for: MicroRNA-9 restrains the sharp increase and boost apoptosis of human acute myeloid leukemia cells by adjusting the Hippo/YAP signaling pathway
Source: Bioengineered. 2021 Jun 24;12(1):2906–14. doi: 10.1080/21655979.2021.1915727 (PMC8806226; doi:10.1080/21655979.2021.1915727)

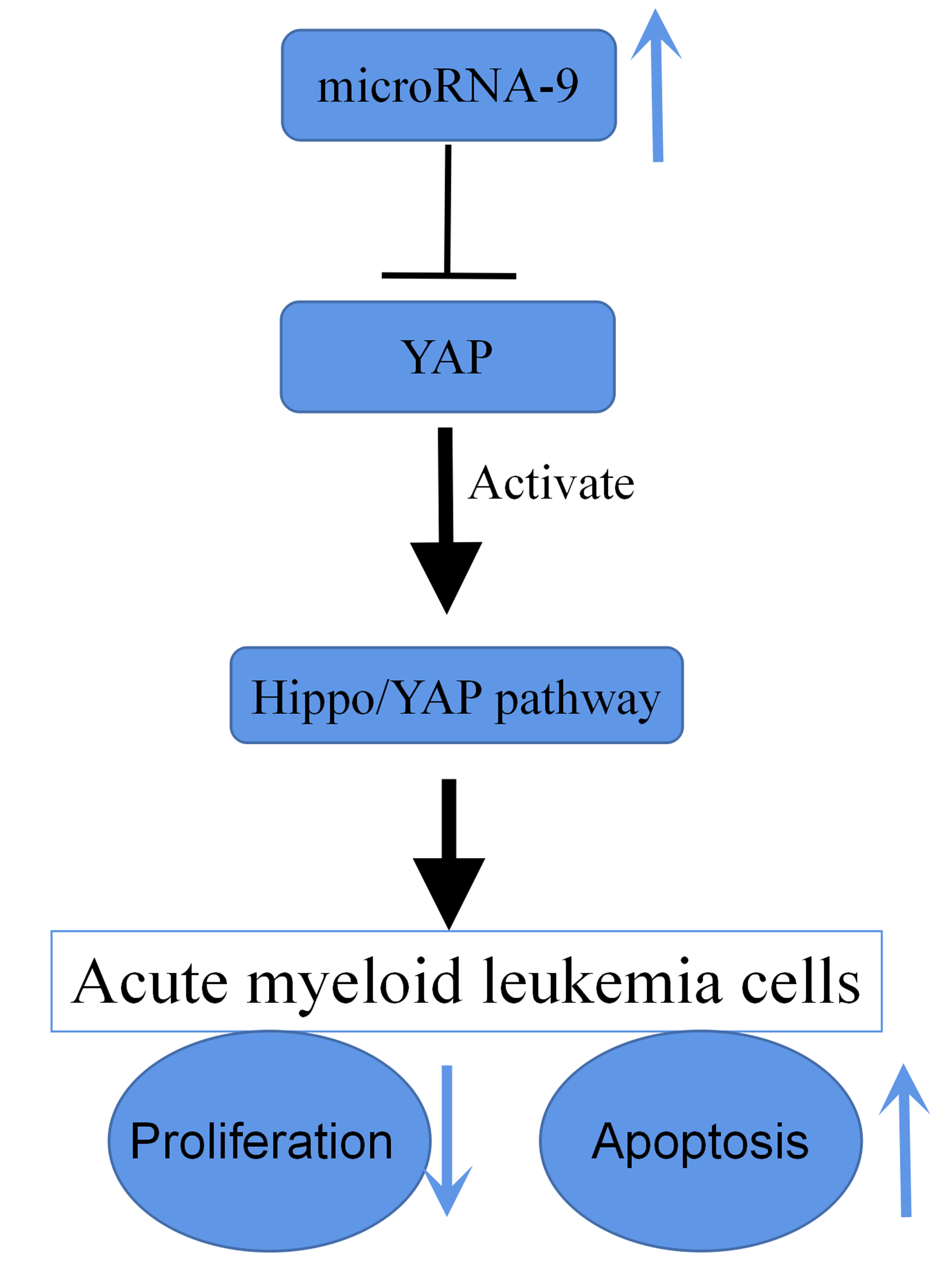

Supplement: Supplemental Material [file KBIE_A_1915727_SM7562.tif]
